# Zona de l’enfant: à propos de deux cas

**DOI:** 10.11604/pamj.2019.32.199.15403

**Published:** 2019-04-24

**Authors:** Fatima-Zahra Agharbi

**Affiliations:** 1 Hôpital Civil Tétouan, Maroc

**Keywords:** Enfant, zona, nourisson, Child, herpes zoster, newborn

## Abstract

Le zona de l'enfant est une situation rare mais souvent bénigne et ne nécessitant qu'un traitement symptomatique. Les antiviraux sont exceptionnellement utilisés devant les formes compliquées qui sont l'apanage surtout des immunodéprimés ou de localisation ophtalmique. Pour le nourrisson, il s'agit le plus souvent d'une contamination in utero au cours d'une varicelle maternelle. Nous rapportons deux cas de Zona chez des enfants immunocompétents. Le premier chez un nourrisson et le deuxième chez un enfant de 7 ans.

## Abstract (en)

Herpes zoster is a rare, usually benign disease in children, only requiring symptomatic treatment. Antivirals are used under exceptional circumstances in patients with complex forms of herpes zoster, usually affecting immunocompromised persons, or with ophthalmic involvement. In newborns, it is more often due to intrauterine contamination during maternal varicella. We report two cases of herpes zoster in immunocompetent children. The first case involves a newborn while the second a 7-year old child.

## Introduction

Le zona survient par réactivation du virus VZV (virus varicelle-zona) resté quiescent dans les ganglions sensitifs dorsaux après la varicelle. Chez l'enfant il se manifeste souvent à côté de l'atteinte cutanée classique, par des signes généraux importants. Son évolution est en général favorable, les algies post zostériennes restent exceptionnelles. Les formes de l'enfant immunodéprimé peuvent mettre en jeu le pronostic vital et imposent un traitement spécifique. La prévention par la vaccination ne parait pas applicable et semble augmenter l'incidence de la varicelle chez l'adulte potentiellement grave [1].

## Patient et observation

**Figure 1:**
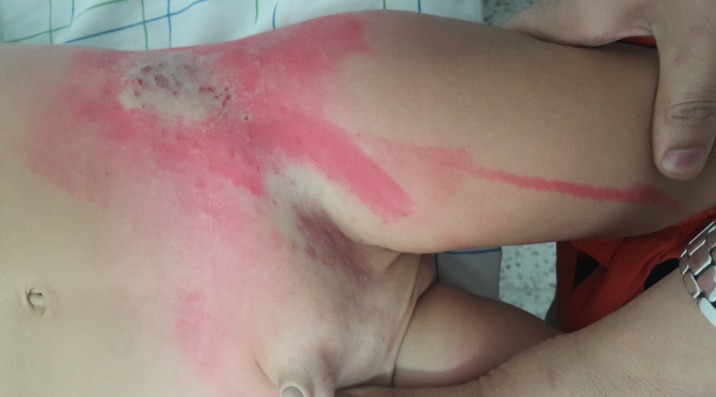
Vésicules groupées en bouquet le long du métamère L1

**Observation 1:** Nourrisson de 6 mois ayant comme antécédent une varicelle chez la maman à 7 mois de grossesse qui présentait depuis 03 jours des lésions cutanées asymptomatiques. L'examen dermatologique trouvait des vésicules groupées en bouquet avec une disposition métamérique le long du métamère L1 (Figure 1). Le reste de l'examen somatique était sans anomalies. Le diagnostic de Zona a été retenu devant l'aspect clinique typique. Devant le terrain immunocompétent, le nourrisson a été mis sous soins locaux et crème cicatrisante. L'évolution était favorable avec cicatrisation au bout d'une semaine.

**Figure 2:**
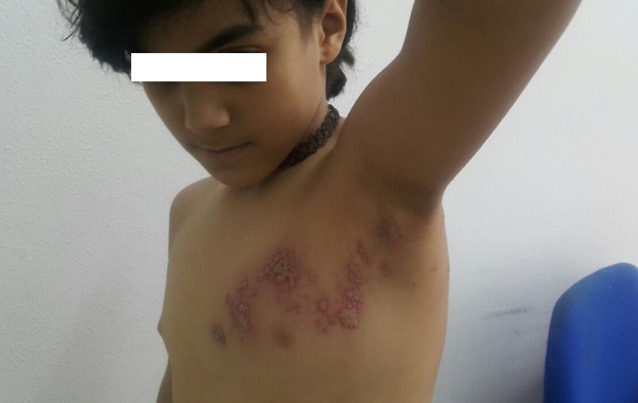
Vésicules groupées en bouquet le long du métamère D4

**Figure 3:**
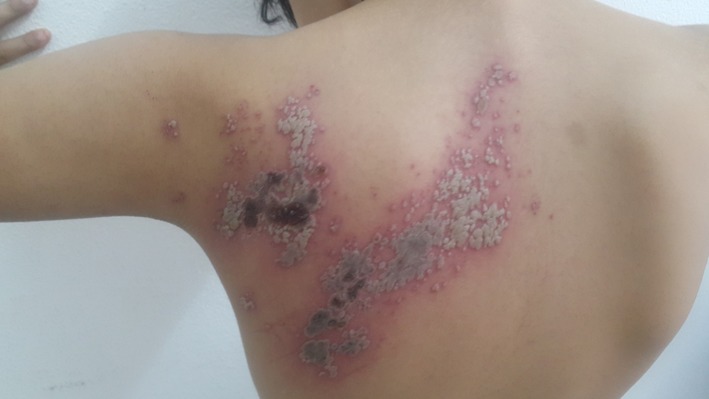
Vésicules groupées en bouquet nécrotiques le long du métamère D4

**Figure 4:**
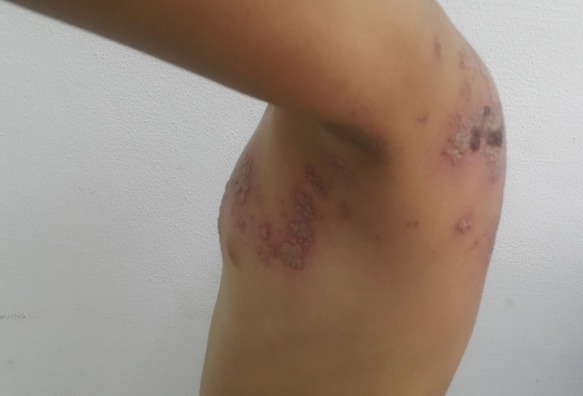
Vésicules groupées en bouquet nécrotiques le long du métamère D4

**Observation 2:** Enfant de 8 ans, notion de varicelle à l'âge de 3 ans qui présente depuis une semaine des lésions cutanées avec sensation de picotement. L'examen trouvait des vésicules groupées en bouquet nécrotiques par endroits à disposition métamérique le long du métamère D4 (Figure 2, Figure 3, Figure 4). Vu l'absence de terrain d'immunodépression, le traitement était symptomatique avec une bonne évolution.

## Discussion

L'homme est le réservoir exclusif du virus varicelle- zona (VZV), virus neurotrope ayant la particularité de rester quiescent dans les ganglions sensitifs dorsaux. Le VZV appartient à la famille des *Herpès viridae*, virus à enveloppe, avec génome à ADN. Il a une affinité particulière pour la peau, le système nerveux et les poumons [2]. Après la primo-infection (varicelle), le virus gagne les ganglions sensitifs par voie hématogène et/ou neurogène à partir de la peau ou les muqueuses. Lors de la réactivation, il migre le long des fibres nerveuses sensitives jusqu'à la peau. Une éruption vésiculeuse caractéristique apparait alors dans le métamère correspondant au ganglion rachidien colonisé lors de la primo-infection. Sur le plan immunologique, il semble que l'immunité humorale permette de contenir un VZV réactivé à un seul dermatome comme le montre l'ascension des anticorps anti- VZV de type IgA et IgG. L'immunité cellulaire intervenant dans le maintien de la phase de latence. La contamination se fait par contact direct avec un sujet présentant une varicelle (gouttelettes respiratoires ou liquide des vésicules) ou un sujet présentant un zona (liquide des vésicules). La réactivation est favorisée par l'immunodépression. La majorité des cas de zona de l'enfant surviennent après l'âge de 5 ans, Parmi tous les cas rapportés de zona, moins de 10% ont moins de 20 ans, et 5% ont moins de 15 ans [3], et une incidence de 0,74 pour 1000 chez la population de moins de 9 ans [4]. 26 cas de zona du nourrisson ont été rapportés dans la littérature. Parmi ces cas, 15 semblent issus d'une exposition intra-utérine et 6 cas n'avaient aucune histoire d'exposition antérieure au virus [5, 6]. La prise en charge fait appel d'abord aux bains quotidiens, des soins locaux par un antiseptique, L'aciclovir par voie orale constitue le traitement de première intention à raison de 20 mg/Kg/8h pendant 5 à 7 jours. Les autres antiviraux tels le famciclovir et le valacyclovir ne sont pas indiqués chez le nourrisson [7]. Le pronostic excellent, Les complications les plus fréquentes sont une surinfection bactérienne secondaire, une dépigmentation et des cicatrices, d'autres complications sont plus rares tels une encéphalite, une ventriculite, une sclérokeratite et une uvéite antérieure [7].

## Conclusion

Le zona est une affection rare chez l'enfant, d'évolution le plus souvent favorable sans séquelle, en particulier douloureuse. Il ne nécessite pas de traitement spécifique en dehors des formes ophtalmiques, des formes compliquées ou en cas de terrain immunodéprimé. Son diagnostic est essentiellement clinique. L'élargissement de la vaccination à la population générale ne s'impose pas car il expose au problème de l'augmentation de nombre des femmes en âge de procréer séronégatives.

## Conflits d’intérêts

L'auteur ne déclare aucun conflit d'intérêts.

## References

[1] Lethel V, Mancini J (2002). Le zona de l'enfant. J Pédiatr Puériculture.

[2] Fillet AM, Lebon P (1999). Virus de IO varicelle et du zona ln: Denis F Les virus transmissibles de la mère à I'enfant.

[3] Teran CG, Villarroel P, Teran-Escalera CN (2008). Herpes zoster in healthy children. Int J Infect Dis.

[4] Kakourou T, Theodoridou M, Mostrou G (1998). Herpes zoster in children. J Am Acad Dermatol.

[5] Kashima M (2003). A rather rare encounter with herpes zoster in a male infant. J Dermatol.

[6] Brar BK, Pall A, Gupta RR (2003). Herpes zoster neonatorum. J Dermatol.

[7] Leung AK, Robson WL, Leong AG (2006). Herpes Zoster in Childhood. J Pediatr Health Care.

